# CHANGES IN RELIGIOUSNESS AMONG OLDER ADULTS FOLLOWING CANCER DIAGNOSIS

**DOI:** 10.1093/geroni/igz038.2377

**Published:** 2019-11-08

**Authors:** Tonia K Murphy, Sara Wilton, Amanda Blackmar, Edward H Thompson, Andrew Futterman

**Affiliations:** 1 Loyola University of Maryland, Baltimore, Maryland, United States; 2 College of the Holy Cross, Worcester, Massachusetts, United States; 3 Department of Psychology, Loyola University Maryland, Baltimore, Maryland, United States

## Abstract

This study aims to identify the changes in individuals’ religious beliefs after receiving a cancer diagnosis. Open-ended religious development interviews were completed with 278 older adults (aged 55 to 102) in the northeast United States. Of these 278, 16 of the interviewees had received a cancer diagnosis within the previous two years. These older adults who report a cancer diagnosis describe few changes in their overall religiousness. On the contrary, these individuals describe longstanding, generally consistent patterns of religiousness that began early in life, most often related to their family upbringing. While most of these cancer-diagnosed individuals have sustained their religious involvement over time, a few have rejected or substantially modified their religious involvement for reasons unrelated to their recent diagnosis (e.g., most often due to marrying someone of a different faith tradition). In addition, those who received a cancer diagnosis describe their religious involvement primarily in terms of its emotional and social reward. These individuals report little complexity of religious belief - little questioning, little doubt in religious doctrine. Instead they report a straightforward, deep, comforting religious belief. This is in contrast to frequent critical comments on religious belief and behavior reported by healthy older adults in our sample. Such findings are in keeping with previous research that describes a more extrinsically oriented religious involvement among older adults who are ill or vulnerable. We discuss these findings in light of both social selectivity theory (E.g., Carstensen, 1992) and stress and coping theory (Lazarus & lazarus, 2006).

